# Two Examples of RNA Aptamers with Antiviral Activity. Are Aptamers the Wished Antiviral Drugs?

**DOI:** 10.3390/ph13080157

**Published:** 2020-07-22

**Authors:** Alfredo Berzal-Herranz, Cristina Romero-López

**Affiliations:** Instituto de Parasitología y Biomedicina López-Neyra, (IPBLN-CSIC), Av. del Conocimiento 17, PTS Granada, Armilla, 18016 Granada, Spain; cristina_romero@ipb.csic.es

**Keywords:** aptamers, antiviral RNAs, viral RNA genome, functional RNA domains, RNA tools, RNA structure/function

## Abstract

The current Covid-19 pandemic has pointed out some major deficiencies of the even most advanced societies to fight against viral RNA infections. Once more, it has been demonstrated that there is a lack of efficient drugs to control RNA viruses. Aptamers are efficient ligands of a great variety of molecules including proteins and nucleic acids. Their specificity and mechanism of action make them very promising molecules for interfering with the function encoded in viral RNA genomes. RNA viruses store essential information in conserved structural genomic RNA elements that promote important steps for the consecution of the infective cycle. This work describes two well documented examples of RNA aptamers with antiviral activity against highly conserved structural domains of the HIV-1 and HCV RNA genome, respectively, performed in our laboratory. They are two good examples that illustrate the potential of the aptamers to fill the therapeutic gaps in the fight against RNA viruses.

## 1. Introduction

The current global pandemic caused by SARS-Cov-2 has revealed the fragility of our global health security system. Researchers have been warning governments about the risk of different heath threats we face today, and this outbreak has clearly shown that no country, not even the so called first world, is safe and protected against a sanitary emergency. Among other deficiencies, one of the major limitations to fight against the current viral infection has been the lack of specific therapeutic agents, which is a common fact for many other infections caused by RNA viruses (HIV, HCV, SARS, Ebola, Dengue, and Zika viruses are just few examples of modern viral RNA outbreaks). Researchers have traditionally devoted many efforts to searching for the magic molecule or therapeutic strategy that would allow fighting efficiently against these tiny organisms that so easily put the most evolved living being in check.

**Figure 1:**
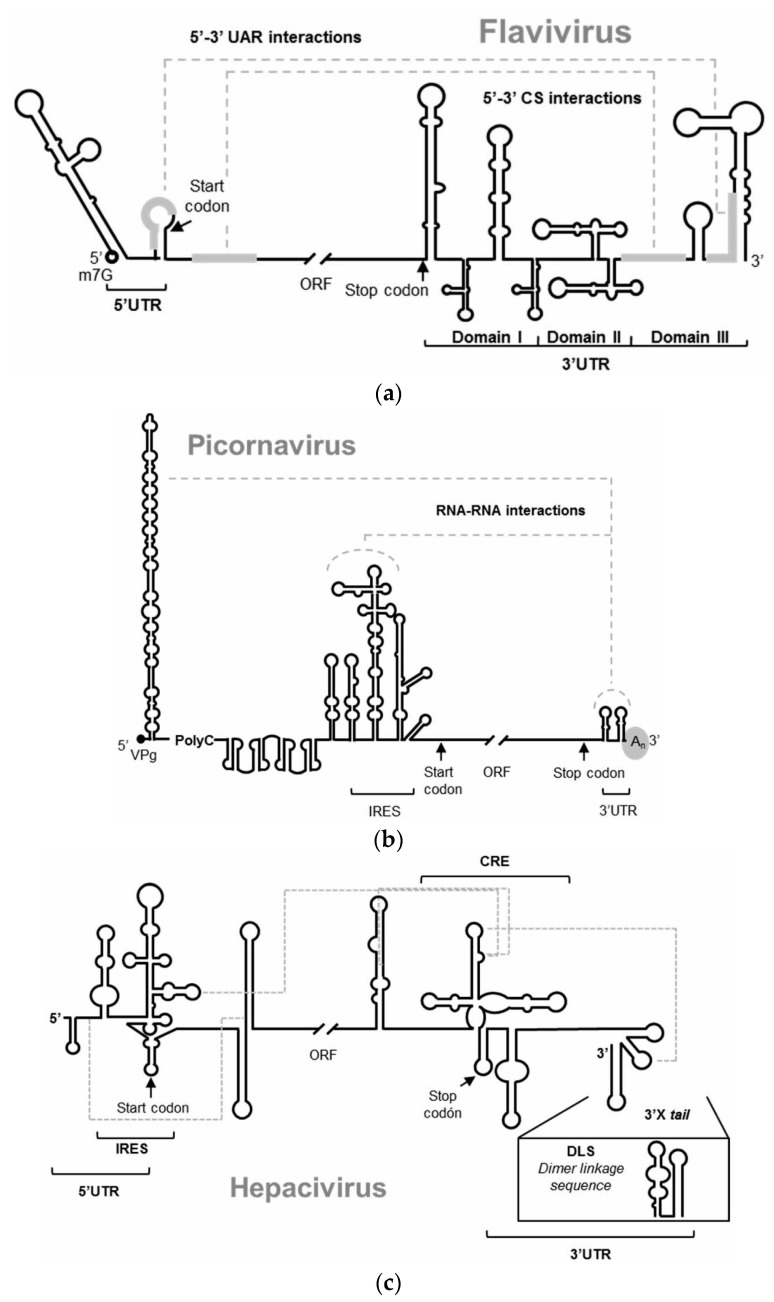
Schematic representation of the 5′ and 3′ ends of the RNA genomes of (**a**) *Flavivirus*, represented by the WNV, (**b**) *Picornavirus*, (**c**) *Hepacivirus*, represented by the HCV. The figures depict the main highly conserved structural RNA elements within their two genomic ends. Defined functional RNA–RNA interactions are indicated with broken gray lines. Translation start and stop codons are indicated by arrows.

RNA viruses store all the required information for the completion of the infectious cycle in their RNA genome. In addition to the information that encodes the viral proteins, the genome includes the information required to efficiently sequester and utilize the cellular machinery and all the information involved in the regulation of the viral processes. RNA viruses have developed different molecular strategies to bear all the information, compacting it in different hierarchical levels of coding within the RNA genome [1,2,3]. Thus, the nucleotide sequence stores essential information in highly conserved structural domains composed of discrete structural units (Figure 1) [1]. This information storage system overlaps and complements the protein coding information. RNA genomes are multifunctional molecules: they act as a replication template, they act as mRNAs, and behave as true non-coding RNAs (ncRNAs), playing several essential functions for the viral cycle and the regulation of viral processes [1]. Elucidating the function of each RNA domain and their modus operandi would provide an excellent scenario to address the control of infections caused by RNA viruses, broadening beyond proteins the repertoire of potential target candidates to fight viral diseases. Interfering with the structure of the functional genomic RNA domains or compromising their mechanisms of action should challenge their proper functioning and therefore the completion of the viral propagation cycle. This report shows how aptamers can exploit the structural features of viral genomic RNAs as therapeutic targets, while allowing the implementation of therapeutic tools, which may be developed against a variety of RNA viruses.

## 2. Aptamers, Selection Procedure, and Features

It was in 1990 that two independent publications from the groups of Jack Szostack and Larry Gold set the basis for the development of the currently known aptamer technology [4,5]. The group of Jack Szostak described the isolation of short RNA oligonucleotides that were able to efficiently bind to organic dyes. They coined the term aptamer to name these binder molecules [4]. On the other hand, the principles of the general strategy for the selection of aptamers that is known as SELEX were defined by the Larry Gold’s group. SELEX stands for systematic evolution of ligands by exponential enrichment [5]. Aptamers are defined as short oligonucleotides, DNA and RNA, that exhibit extraordinary binding efficiency to a specific target molecule [4]. Since then, numerous reports have described the successful identification of aptamers targeting from single molecules like ions, amino acids, nucleotides, antibiotics, etc., to macromolecules like peptides, proteins, or nucleic acids, and even targeting viruses and full cells [6,7,8,9,10,11,12,13,14]. 

The potential of this technology was envisioned quite early, and the therapeutic application of aptamers for a wide variety of diseases was proposed. It was in 2004 that the FDA approved the first aptamer to be used as a specific drug. This aptamer, which was named Pegaptanib and commercialized as MACUGEN, was meant for the treatment of the wet age-related macular degeneration [15,16,17]. Nevertheless, the full therapeutic potential of the aptamers is still to be developed.

### 2.1. SELEX

**Figure 2:**
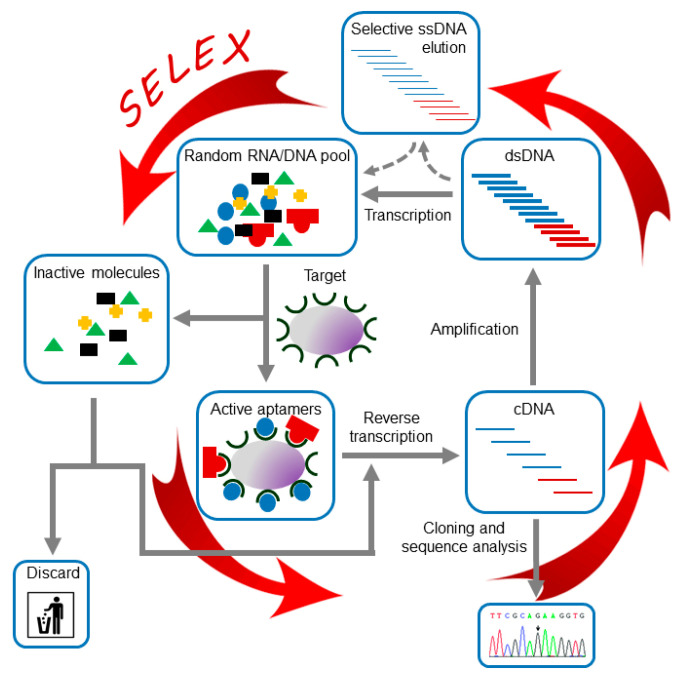
SELEX procedure. The figure shows a general scheme of the RNA aptamer in vitro selection process. Broken lines indicate the alternative steps required for the selection of DNA aptamers.

Independently of the application of the aptamers and the nature of their target, all reported aptamers have been identified following a common experimental strategy known as SELEX [5,18], which is outlined in Figure 2. Briefly, a large in vitro synthesized population of variable sequence oligonucleotides, typically ranging from 10^12^ to 10^16^ variants, is subjected to the SELEX procedure, which consists of iterative cycles of exposition to the target molecule to allow their binding to the target, separation of those molecules capable of binding and their further amplification. This procedure allows the progressive enrichment of the population in winner molecules for specific binding to the desired target. Those winner molecules are named aptamers. 

The researcher can control at any time the conditions of the selection and recovery steps during the process. Usually, the main goal is to identify the selection to yield aptamers that exhibit high affinity by a desired target, but the researcher can also pursue the selection of aptamers that achieve stable binding by the imposed conditions. It has been shown that aptamers may bind the ligand molecule with efficiencies that even may exceed that of antibodies for their antigens [19].

### 2.2. Aptamer Features

Aptamers show some important advantages [19], including that the use of experimental animals is not required for their production, as opposed to antibodies. They can be chemically modified to increase their stability or resistance to nuclease degradation, by means of the addition of certain chemical groups (fluoro, methyl, or methoxy among them) at different positions of the nucleotide, or by replacing specific nucleotides by nucleotide analogs (e.g., locked nucleic acids-LNAs, peptide nucleic acids-PNAs). Thus, in no case the specificity of the nucleotide sequence of the aptamer is altered. Modifications can be also added to improve specific aptamer delivery to the target. Aptamers can also be modified by incorporating different types of labeling (e.g., radioactive, fluorescent) and various functional groups, as well as to conjugate with other molecules (PEG, sugar residues, siRNAs, among others) to achieve many other different functionalities [20]. 

Specificity of aptamers is determined by both their sequence and their three-dimensional folding. Further, aptamers recognize specific functional groups of the target molecule in a precise spatial distribution. Therefore, aptamers are good candidates to target complexly folded targets. Consequently, in contrast to other nucleic acid based strategies (ribozymes, antisense oligonucleotides, siRNA, among others), aptamers are postulated as excellent candidates to directly interfere with the functioning of essential structural domains of viral RNA genomes.

## 3. Antiviral Aptamers

Targeting viral proteins is the common strategy that has been followed in most attempts aimed to develop efficient antiviral agents regardless of the drug to be used. Aptamers selected against a variety of viral proteins have also been reported in the literature. Those studies account for the achievement of a range of antiviral activity levels [18]. 

Unfortunately, up to now such a strategy has been found to be very inefficient, yielding in most cases unacceptable therapeutic results. Even in the best scenarios, the achievements that have occurred over the years of enormous economic and human resources investments are unfortunately not enough to compensate for the unacceptable number of human lives and the consequences on the health and well-being of humans that viral infections take, among other consequences. This is evident in its maximum expression in a pandemic situation like the one we are currently experiencing. Therefore, it is widely accepted by the scientific community the need of exploring alternative viral targets and developing of new therapeutic antiviral strategies [1]. Targeting the essential information encoded in conserved structural RNA domains in viral genomes represents an attractive yet unexplored strategy. Nucleic acids are shown as the most likely suitable tools to interfere directly with such information, aptamers being excellent candidates for challenging the functioning of structured RNA molecules. Herein, we present an extension of a video we communicated at the 5th ECMC describing two examples of selection and characterization of aptamers targeting essential structural domains of respective viral RNA genomes, performed in our laboratory [21].

**Figure 3:**
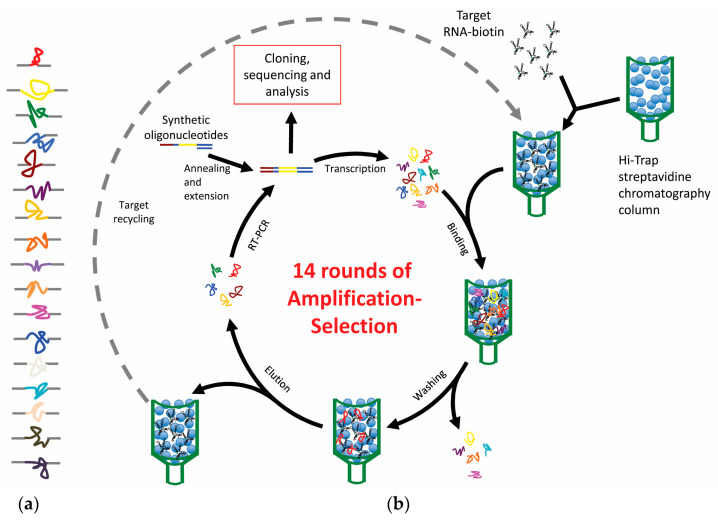
Schematic representation of the SELEX procedure we applied for the isolation of aptamers against the HIV and HCV genomic RNA fragments. (**a**) Representation of the starting population of RNA oligonucleotides that is subjected to the selection process. The colored line represents the 25–30 nt-long random sequence region. The different folding determined by the different sequence is depicted by the free hand drawing. Gray lines represent the fixed flanking sequences. (**b**) SELEX procedure. Details of the protocol are provided in [24,25].

In both cases we followed a common SELEX strategy (Figure 3). Briefly, the target subgenomic RNA fragment was internally biotinylated during in vitro synthesis, under experimental conditions to render a theoretical incorporation of one biotinylated-UTP residue per RNA molecule [22,23]. The low rate of nucleotide modification was imposed to the lowest, to achieve the minimum possible interference in the proper folding of the target viral RNA by the bulky biotin residue. Then, the target was immobilized to a sepharose-streptavidin column, allowing it to adopt its natural conformation. In parallel, the starting pool of RNA oligonucleotides resulting from the randomization of 25–30 consecutive nucleotides flanked by fixed sequences was synthesized [24]. Theoretically, each RNA oligonucleotide contains a different sequence, which will determine a different molecular folding. The combination of the sequence and the structure will be responsible for the functioning of each molecule, determining whether a specific oligonucleotide is capable of binding to the target RNA molecule.

The pool of RNA oligonucleotides was binding-challenged against the immobilized target molecule. Then, only oligonucleotides capable of binding to the target RNA were retained. After their selective elution and amplification by RT-PCR, the new generated pool was subsequently introduced in a new round of selection. This was repeated until rendering the most efficient binders to the viral genomic fragment [24]. 

### 3.1. Anti HIV-1 RNA Aptamers

**Figure 4:**
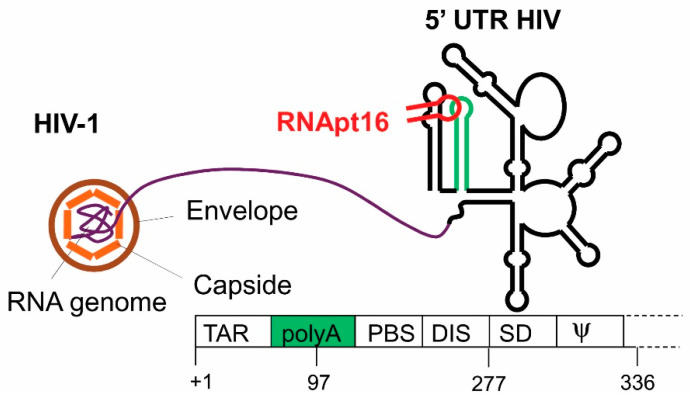
Representation of the HIV-1 particle. The secondary structure model of the genomic RNA fragment used as the aptamers’ target in the selection procedure is outlined on the right, depicting the well characterized functional structural elements. The genomic organization of these structural elements is indicated at the bottom. The polyA domain is indicated in green. The designed RNApt16 aptamer targeting the apical loop of the polyA element is represented in red.

We applied the above experimental strategy to isolate aptamers against the 5′UTR of the human immune deficiency type 1 virus (HIV-1) [25]. For this purpose, the target RNA consisted of the genomic viral RNA fragment comprising the first 308 nt of its 5′UTR. This fragment is common to all genomic and subgenomic HIV-1 RNAs [26]. It contains several well defined structural elements whose functioning is essential for the HIV efficient infection cycle (Figure 4).

The starting RNA pool consisted of theoretically 10^15^ oligonucleotide variants, resulting from the randomization of 25 contiguous nucleotides. The selection cycle was repeated up to 14 times [25]. The sequence analysis of resulting molecules revealed the presence of the eight nt-long sequence, 5′-GGCAAGGA-3′, in the approximately 90% of the selected sequences (Table 1). Interestingly, this octanucleotide is complementary to an eight nt-long sequence within the apical loop of the polyA structural element of the HIV-1 5′UTR. This result points to the polyA domain as the target of the resulting aptamers [25].

**Figure 5:**
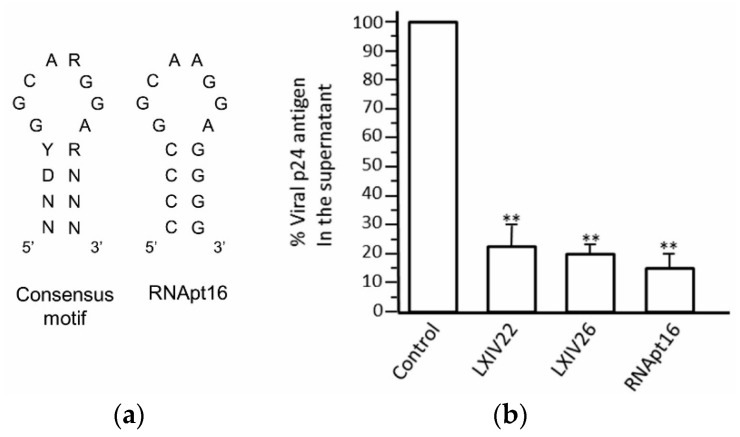
Inhibition of HIV-1 particles production by the RNApt16 aptamer. (**a**) Sequence and secondary structure of the 16 nt-long consensus selected structural motif and the designed RNApt16 aptamer. R, purine; Y, pyrimidine; D; A, G, or U; N, any ribonuleotide. (**b**) Inhibition of HIV-1 particles production assays. Inhibitory efficiency of RNApt16 aptamer was measured as p24 antigen production. Its activity was compared with that showed by the most frequent aptamers resulting from the SELEX procedure LXIV22, LXIV26. Data represent the mean of three independent assays. **, significant differences as compared to the control (*p* < 0.01). Figure adapted from [27].

Instead of concluding the aptamer’s selection at this point, we decided to introduce an innovative further step. We complemented the SELEX approach with a bioinformatics analysis of the identified sequences [25]. It consisted of a thorough comparison of the sequences derived from each selection round and their respective secondary structure folding, determining the sequence and structural evolution of the resulting molecules along the process. This allowed us to identify a minimal 16 nt-long structural element common to most of the selected molecules from round 11 to 14. Interestingly, this 16 nt-long structural motif contained the above mentioned octanucleotide sequence motif universally exposed in an apical loop closing a 4 bp stem of variable sequence (Figure 5). These results indicated that the properly folded 16 nt-long stem-loop element containing the 5′-GGCAAGGA-3′ sequence motif correctly exposed was responsible for the aptamers’ activity. This hypothesis was confirmed by targeting the HIV-1 5′UTR fragment with a synthetic 16 nt-long RNA molecule (RNApt16) comprising the minimal selected structural RNA. First RNApt16 showed binding affinity and efficacy in a similar extend to that shown for the parental aptamers [25].

Inhibition studies of HIV-1 particles production in a cell culture analysis, by measuring the p24 viral antigen, demonstrated that RNApt16 yielded inhibition levels higher than 85%, achieving higher inhibition ratios than the two most abundant parental aptamers (XIV22 and XIV26) (Figure 5) [25].

The innovative combination of a SELEX procedure with a bioinformatics analysis allowed the identification of a common 16 nt-long structural element, consisting of an octanucleotide of fixed sequence exposed in a loop motif closed by 4 bp stem of variable sequence that retained the full antiviral activity. It served as base for the design of the, to our knowledge, smallest aptamer ever described. It is worth noting that the minimum molecular size of the aptamers is an important limitation. The size is imposed by the need of carrying flanking fixed sequences required for the amplification steps of the SELEX procedure. The application of the bioinformatics analysis provides unique means of identifying the minimum domain responsible of the entire aptamer activity.

### 3.2. Anti HCV RNA Aptamers

The hepatitis C virus (HCV) has been another favorite virus for the development of nucleic acids-based antiviral strategies. The organization and structure of its RNA genome have been extensively studied [28]. It contains numerous structurally conserved elements that play essential roles in the viral infection cycle (Figure 1). Among them, the so called cis-replication element (CRE), located at the 3′ end of the single open reading frame of the viral genome, has been identified as a center of regulation of major viral processes (e.g., replication, translation, genome dimerization) [29]. Due to its functional importance for the establishment of the viral infection, it represents a good potential antiviral target.

**Figure 6:**
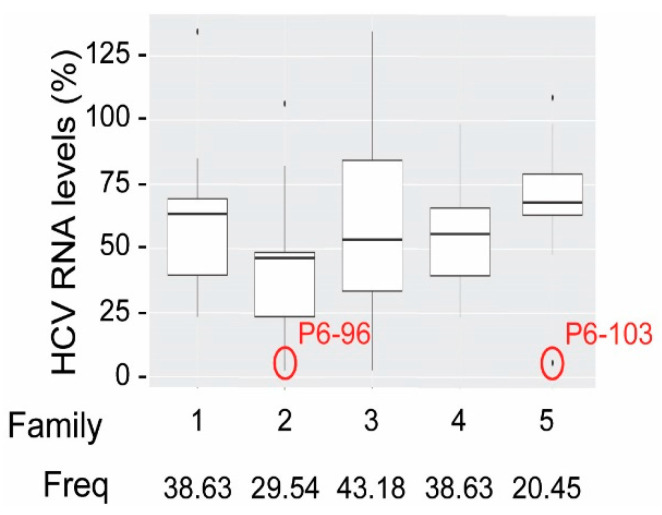
Inhibition of HCV replication in Huh-7 cells. Graph shows the HCV RNA levels in a hepatoma cell line supporting the autonomous cytoplasmic replication of a HCV subgenomic construct. Cells were transfected with the selected aptamers and HCV RNA levels were detected by RT-qPCR 24 h after transfection. Boxplot was generated with these data in order to group those aptamers with common sequence motifs, belonging to each of the identified families (1 to 5). The box reflects the 1st and 3rd quartiles, and the median is represented by a black line within the box for each aptamer family. Freq, refers to the prevalence of each family within the aptamer population.

We applied the previously described SELEX strategy to identify RNA aptamers against a 194 nt-long subgenomic RNA comprising the CRE [24]. A population of theoretically more than 10^18^ RNA variants, resulting from the randomization of a stretch of 30 nts, was introduced in the selection process. The resulting variant molecules after six selection rounds were classified in up to five families, defined by common selected sequence motifs. As expected, each common sequence possessed its complementary counterpart within the viral CRE [24]. The prevalence of each common sequence motif in the final population could be related to a direct participation in the interaction of the aptamer with the CRE target or to a stabilization role of the aptamer:CRE complex. Interestingly, many of the selected aptamers even bore sequence motifs belonging to different families, reinforcing the idea that each sequence family accomplished different functions [30]. The antiviral potential of the selected aptamers was determined by their ability to inhibit viral replication, using a subgenomic HCV replication model in Huh-7 cell line culture. Results demonstrated that aptamers belonging to family 2 promoted a slightly more potent inhibitory effect on HCV replication levels (Figure 6). Nevertheless, the inhibitory activity of the different aptamer families was quite similar, suggesting that every sequence motif must be preserved to achieve the aptamer function. Importantly, two aptamers, P6-96 and P6-103, significantly reduced the HCV RNA levels up to 95% (Figure 6) [30,31]. Interestingly, the more efficient inhibitors putatively target the essential 5BSL3.2 component of the CRE [30].

In an independent series of experiments, we isolated a new collection of RNA aptamers conjugated with ribozymes that specifically targeted the translation-essential HCV IRES (internal ribosome entry site), which is located at the 5′ end of the RNA genome [23]. Those RNA aptamers exhibited up to 90% viral translation inhibition rates [23,32,33,34,35].

All together the above reported results point the great antiviral potential of targeting essential structural RNA domains of the HCV genome.

## 4. Conclusions

Aptamers offer a potential means for the development of efficient antiviral drugs. Their mechanism resides in the recognition of functional groups in a specific structural context in the target molecule, similar to antigens recognition by antibodies. Viral RNA genomes store essential information for the completion of the viral propagation cycle in structural RNA elements. These elements are grouped in structurally complex domains and regions that coincide with the highest conserved regions of the highly variable viral genome among different viral isolates. The preservation of their structure is essential for the proper functioning of each of these elements, offering an excellent scenario for fighting infections caused by RNA viruses. Aptamers have proven to be capable of interfering with the activity of these essential domains by competing with the interactions they are involved in or by modifying their structure. Different groups have already reported aptamers that induce potent inhibition rates of a variety of RNA viruses by targeting conserved genomic RNA domains. Therefore, aptamers emerge as real molecular tools to fight viral infections.

## Figures and Tables

**Table 1:** Selected antiHIV-1 aptamers after 14 rounds of selection.

Nº Repetitions	Aptamer ^1^
**23**	CACCACUAUUGUU**GGCAAGGA**AGCA
**6**	GUAC**GGCAAGGA**GUACAUCGUAGCA
**2**	CACAACCUGGGU**GGCAAGGA**ACCCA
**1**	CACCGCUAUUGUU**GGCAAGGA**AGCA
**1**	CACCACUAUUGUU**GGCAAGGA**AGCA
**1**	GUAC**GGCAAGGA**GUACAUCGCAGCA
**1**	CACUACUCUACGGCUCGAAGCCCCA
**1**	AACCACAACGGCUAACCACUGCCCA
**1**	CACUACCGACCGUCCACACCAGCCA

^1^ Sequence corresponding to the 25 nt-long variable region is shown. Consensus octanucleotide complementary to the HIV-1 polyA element is shown in bold. Underlined sequences correspond to motifs complementary to other 5′UTR HIV-1 sequences.

## References

[1] Romero-López C., Berzal-Herranz A. (2013). Unmasking the information encoded as structural motifs of viral RNA genomes: A potential antiviral target. Rev. Med. Virol..

[2] Moomau C., Musalgaonkar S., Khan Y.A., Jones J.E., Dinman J.D. (2016). Structural and functional characterization of programmed ribosomal frameshift signals in west nile virus strains reveals high structural plasticity among cis-acting RNA elements. J. Biol. Chem..

[3] Kendra J.A., Advani V.M., Chen B., Briggs J.W., Zhu J., Bress H.J., Pathy S.M., Dinman J.D. (2018). Functional and structural characterization of the chikungunya virus translational recoding signals. J. Biol. Chem..

[4] Ellington A.D., Szostak J.W. (1990). In vitro selection of RNA molecules that bind specific ligands. Nature.

[5] Tuerk C., Gold L. (1990). Systematic evolution of ligands by exponential enrichment: RNA ligands to bacteriophage T4 DNA polymerase. Science.

[6] Ellington A.D., Szostak J.W. (1992). Selection in vitro of single-stranded DNA molecules that fold into specific ligand-binding structures. Nature.

[7] Schneider D., Tuerk C., Gold L. (1992). Selection of high affinity RNA ligands to the bacteriophage R17 coat protein. J. Mol. Biol..

[8] Wilson D.S., Szostak J.W. (1999). In vitro selection of functional nucleic acids. Annu. Rev. Biochem..

[9] Rajendran M., Ellington A.D. (2008). Selection of fluorescent aptamer beacons that light up in the presence of zinc. Anal. Bioanal. Chem..

[10] Raddatz M.S., Dolf A., Endl E., Knolle P., Famulok M., Mayer G. (2008). Enrichment of cell-targeting and population-specific aptamers by fluorescence-activated cell sorting. Angew. Chem. Int. Ed. Engl..

[11] Torres-Chavolla E., Alocilja E.C. (2009). Aptasensors for detection of microbial and viral pathogens. Biosens. Bioelectron..

[12] Marton S., Reyes-Darias J.A., Sánchez-Luque F.J., Romero-Lopez C., Berzal-Herranz A. (2010). In vitro and ex vivo selection procedures for identifying potentially therapeutic DNA and RNA molecules. Molecules.

[13] Ku T.H., Zhang T., Luo H., Yen T.M., Chen P.W., Han Y., Lo Y.H. (2015). Nucleic acid aptamers: An emerging tool for biotechnology and biomedical sensing. Sensors.

[14] Kumar P.K. (2016). Monitoring intact viruses using aptamers. Biosensors.

[15] Ng E.W., Adamis A.P. (2006). Anti-VEGF aptamer (pegaptanib) therapy for ocular vascular diseases. Ann. N. Y. Acad. Sci..

[16] Ng E.W., Shima D.T., Calias P., Cunningham E.T., Guyer D.R., Adamis A.P. (2006). Pegaptanib, a targeted anti-VEGF aptamer for ocular vascular disease. Nat. Rev. Drug Discov..

[17] Ciulla T.A., Rosenfeld P.J. (2009). Antivascular endothelial growth factor therapy for neovascular age-related macular degeneration. Curr. Opin. Ophthalmol..

[18] Gopinath S.C. (2007). Methods developed for SELEX. Anal. Bioanal. Chem..

[19] Tombelli S., Minunni M., Mascini M. (2005). Analytical applications of aptamers. Biosens. Bioelectron..

[20] Odeh F., Nsairat H., Alshaer W., Ismail M.A., Esawi E., Qaqish B., Bawab A.A., Ismail S.I. (2019). Aptamers chemistry: Chemical modifications and conjugation strategies. Molecules.

[21] Berzal-Herranz A., Romero-Lopez C. (2019). RNA Aptamers: Antiviral Drugs of the Future. In Proceedings of the 5th International Electronic Conference in Medicinal Chemistry, MDPI AG: Sciforum. https://ecmc2019.sciforum.net/.

[22] Theissen G., Richter A., Lukacs N. (1989). Degree of biotinylation in nucleic acids estimated by a gel retardation assay. Anal. Biochem..

[23] Romero-López C., Barroso-delJesus A., Puerta-Fernández E., Berzal-Herranz A. (2005). Interfering with hepatitis C virus IRES activity using RNA molecules identified by a novel in vitro selection method. Biol. Chem..

[24] Marton S., Berzal-Herranz B., Garmendia E., Cueto F.J., Berzal-Herranz A. (2012). Anti-HCV RNA aptamers targeting the genomic cis-acting replication element. Pharmaceuticals.

[25] Sánchez-Luque F.J., Stich M., Manrubia S., Briones C., Berzal-Herranz A. (2014). Efficient HIV-1 inhibition by a 16 nt-long RNA aptamer designed by combining in vitro selection and in silico optimisation strategies. Sci. Rep..

[26] Berkhout B. (2011). HIV-1 as RNA evolution machine. RNA Biol..

[27] Berzal-Herranz A., Romero-López C., Berzal-Herranz B., Ramos-Lorente S. (2019). Potential of the other genetic information coded by the viral RNA genomes as antiviral target. Pharmaceuticals.

[28] Romero-López C., Berzal-Herranz A. (2020). The role of the RNA-RNA interactome in the hepatitis C virus life cycle. Int. J. Mol. Sci..

[29] Romero-López C., Berzal-Herranz A. (2017). The 5BSL3.2 functional RNA domain connects distant regions in the hepatitis C virus genome. Front. Microbiol..

[30] Fernández-Sanlés A., Berzal-Herranz B., González-Matamala R., Ríos-Marco P., Romero-López C., Berzal-Herranz A. (2015). RNA aptamers as molecular tools to study the functionality of the hepatitis C virus CRE region. Molecules.

[31] Marton S., Romero-López C., Berzal-Herranz A. (2013). RNA aptamer-mediated interference of HCV replication by targeting the CRE-5BSL3.2 domain. J. Viral Hepat..

[32] Romero-López C., Díaz-González R., Berzal-Herranz A. (2007). Inhibition of hepatitis C virus internal ribosome entry site-mediated translation by an RNA targeting the conserved IIIf domain. Cell Mol. Life Sci..

[33] Romero-López C., Díaz-González R., Barroso-delJesus A., Berzal-Herranz A. (2009). Inhibition of hepatitis C virus replication and internal ribosome entry site-dependent translation by an RNA molecule. J. Gen. Virol..

[34] Romero-López C., Berzal-Herranz B., Gómez J., Berzal-Herranz A. (2012). An engineered inhibitor RNA that efficiently interferes with hepatitis C virus translation and replication. Antivir. Res..

[35] Romero-López C., Lahlali T., Berzal-Herranz B., Berzal-Herranz A. (2017). Development of optimized inhibitor RNAs allowing multisite-targeting of the HCV genome. Molecules.

